# Glycaemic and insulinemic response to dietary carbohydrates in horses

**DOI:** 10.1186/s13028-016-0244-1

**Published:** 2016-10-20

**Authors:** Christine Brøkner, Dag Austbø, Jon A. Næsset, Dominique Blache, Knud Erik B. Knudsen, Hanne H. Hansen, Anne-Helene Tauson

**Affiliations:** 1 Department of Veterinary Clinical and Animal Sciences, Faculty of Health and Medical Sciences, University of Copenhagen, Grønnegaardsvej 3, 1870 Frederiksberg, Denmark; 2 Department of Animal and Aquacultural Sciences, Norwegian University of Life Sciences, Box 5003, 1432 Ås, Norway; 3 School of Animal Biology, Faculty of Natural and Agricultural Sciences, University of Western Australia, 35 Stirling Highway, Crawley, Perth, WA 6009 Australia; 4 Department of Animal Science, Aarhus University, Blichers Allé 20, 8830 Tjele, Denmark; 5 Department of Large Animal Sciences, Faculty of Health and Medical Sciences, University of Copenhagen, Grønnegaardsvej 2, 1870 Frederiksberg, Denmark

**Keywords:** Horses, Dietary fibre, Glucose, Insulin

## Abstract

**Background:**

Dietary sugar and starch affect plasma glucose and insulin concentrations. Little information is available about the effect of dietary fibre on plasma glucose and insulin concentration. It is hypothesized that different dietary fibre compositions will alter post-prandial glycaemic- and insulinemic index of test meals. The objective was to measure postprandial glucose and insulin concentrations in horses fed meals of different fibre compositions.

**Methods:**

Blood was drawn via jugular vein puncture and the glycaemic and insulinemic index were calculated.

**Results:**

The meal effect on glycaemic and insulinemic response followed the expected pattern, where plasma concentrations increased after feeding and declined after peak concentration. Glycaemic index was 100 (H), 102 (OB), 102 (BB) and 106 (M) and did not differ significantly between meals. Insulinemic index was 100 (H), 140 (OB), 121 (BB) and 125 (M) and did not differ significantly between meals.

**Conclusions:**

In conclusion, meals containing different fibre compositions did not affect the glycaemic- and insulinemic index in horses.

## Background

Horses are hindgut fermenting herbivores well adapted to a high carbohydrate diet consisting primarily of Graminaceae (e.g. grasses and sedges) and some Leguminosae (e.g. herbaceous legumes), which both are rich in different plant fibre fractions [1]. The microbes of the equine hindgut produce fibre-fermenting enzymes, which are needed to degrade fibrous materials to monosaccharides. The microorganisms and the anaerobic environment of the hindgut prevent complete oxidation of the monosaccharides and instead short chain fatty acids (SCFA) are produced. The acids produced are mainly acetic acid, propionic acid and butyric acid, of which propionic acid is an important contributor to the blood plasma glucose concentration through hepatic gluconeogenesis. A systematic correlation between fibre fractions and hindgut propionic acid production was reported in horses [2], where more S-NCP resulted in higher production of propionic acid. Depending on growth rate, climate and time of harvest, grasses can contain up to 18 % non-structural carbohydrates (NSC; i.e. sugar, fructans and starch) [3].

A glycaemic index of human food was developed to classify sugar and starch containing foods based on their potential to raise post-prandial blood glucose concentrations, which could not be predicted by chemical food analysis [4]. A similar classification is relevant for horses, as starch and sugar digestion and glucose metabolism in the horse is similar as to human. Additionally, horses have been shown to suffer some of the same health issues associated with high and fluctuating glucose and insulin concentrations as humans i.e. insulin resistance and obesity [5].

Forages therefore have gluconeogenic properties that contribute to a stable blood plasma glucose concentration. This occurs through either glucose absorption in the small intestine or a continuous supply of glucose through hepatic gluconeogenesis where propionic acid from hindgut fibre fermentation is metabolised into glucose. The objective of the present experiment was to measure postprandial glucose and insulin concentrations in horses fed meals of various carbohydrate compositions. It was hypothesized that the different fibre compositions will alter the post-prandial glycaemic and/or insulinemic index of the test meals.

## Methods

### Experimental procedure

A 4 × 4 Latin Square design was used to quantify the effect of four test meals on post prandial blood plasma glucose and insulin concentrations. This study was part of a main experiment where the horses were fed 1.6 kg dry matter hay 2½ h after the morning meals.

Within each experimental period, a sequence of 21 days adaptation to the diets was followed by four sampling days. On each of the four sampling days blood was collected from one horse, resulting in four replicates for each diet. On the blood collection day (day 22 through day 25), the horses were fed the test meal at 6 a.m. and given 30 min to finish the meal. The horses were fasted 8 h prior to the test meal.

### Animals

Four geldings of Norwegian Cold-blooded Trotter horse (age 5–16 years) with an average body weight of 544 kg and a mean body condition score of 6 [6] were used in the experiment. The horses were housed under the same conditions in individual 3 × 3 m stalls on wood shavings. The horses were cared for according to laws and regulations controlling experimentation on live animals in Norway i.e. the Animal Protection Act of (December 20 1974) and the Animal Protection Ordinance concerning Experiments on Animals (December 15 1996).

### Test meals

The composition and intake of carbohydrates of the test meals are shown in Table 1. A detailed description of the chemical composition of the individual ingredients is found in [3]. The test meals consisted of timothy hay (Phleum L.) (H), whole oats (Avena sativa L.) and soaked molassed sugar beet pulp (Beta vulgaris) (Betfor^®^) (OB), whole barley (*Hordeum vulgare* L.) and soaked Betfor^®^ (BB), commercial muesli feed (Equigard^®^) (M). The test meals were formulated to provide the same amount of dry matter and to contain a maximum of 0.54 kg sugar and starch, equivalent to 1 g starch and sugar per kg BW per meal per horse.

**Table 1:** Dietary composition and intake of carbohydrates of the different test meals

	Test meals			
	H	OB	BB	M
Hay, kg DM	1.1			
Oats, kg DM		1.1		
Barley, kg DM			0.8	
Betfor^®^, kg DM		0.3	0.3	
Equigard^®^, kg DM				1.7
Feed intake, kg DM	1.1	1.4	1.1	1.7
*Total carbohydrate intake, kg*				
Sugar	0.12	0.09	0.08	0.27
Starch	–	0.42	0.42	0.08
Total NSP	0.57	0.43	0.26	0.61
-S-NCP	0.02	0.09	0.10	0.19
*Fibre*				
Dietary fibre	0.70	0.52	0.30	0.75
NDF	0.69	0.42	0.21	0.51

*NSP* non-starch polysaccharides, *S-NCP* soluble non-cellulosic polysaccharides, *H* Hay, *OB* Oats and Betfor^®^, *BB* Barley and Barley and Betfor^®^, *M* Equigard^®^

### Sampling of blood

Blood was drawn via jugular vein puncture into heparinized vacutainer tubes at time 0 before the test meal and again at time (min) 30, 45, 60, 75, 90, 105, 120, 135, 150, 165, 180, 210, 240, 270, 330, 390, 450, 510, 570 post feeding. Immediately after sampling, the tubes were centrifuged at 2016*×g* at 4 °C for 10 min Plasma was harvested and stored in polypropylene micro tubes at −20 °C until analysis.

### Chemical analyses of feedstuffs

All feed samples were analysed in duplicate and the obtained mean values reported. Dry matter was determined on original unprocessed feed samples, collected daily in the stall. NDF content with amylase pre-treatment and corrected for ash was analysed by the use of the Fibertec system (Fibertec™ 2010 Auto Fibre Analysis System, Hillerød, Denmark). Starch was analysed by an enzymatic-colorimetric method. Low molecular weight sugars (glucose, fructose and sucrose) were determined by use of two connected enzymatic reactions using hexokinase and glucose-6-phosphate dehydrogenase [3]. Total non-starch polysaccharides (NSP) consist of various fibre fractions of which the soluble non-cellulosic polysaccharides (S-NCP) were determined by gas–liquid chromatography for neutral sugars and by a colorimetric method for uronic acids in three parallel runs. Dietary fibre (DF) was calculated as described in details [3].

### Analytical procedures for glucose and insulin

Blood plasma glucose was determined according to standard procedures (Siemens Diagnostics^®^ Clinical Methods for ADVIA 1650). All analyses were performed using an auto-analyser, ADVIA 1650^®^ Chemistry System (Siemens Medical Solutions, Tarrytown, NY 10591, USA). Intra- and inter assay precision were in all instances below 3 % (CV) and 5 %, respectively. The accuracy was within 4 %. Plasma insulin was assayed in duplicate by a double-antibody radioimmunoassay (RIA). The measurements were made using bovine insulin, the first antibody being raised in guinea pigs and the second in goats. All samples were processed in a single assay and the detection limit was 0.4 µU/ml. Six replicates of three control samples containing 2.19, 4.48, and 11.35 µU/ml were included in the assay and were used to estimate the intra-assay coefficients of variation of 5.5, 5.1 and 6.9 %, respectively. The assays were validated for horse: serial dilutions of horse plasma containing high concentrations of insulin produced curves parallel to the standard curve for the actual hormone [7].

### Calculations

The glycaemic and insulinemic responses are defined by the increment area under the plasma vs. time curve (above pre-feeding plasma concentration) elicited by a meal. Area under the plasma glucose (AUC_glucose_) and insulin (AUC_insulin_) curve was calculated by the trapezoidal method of numerical integration [4]. Additionally the responses were grouped in time-interval in order to evaluate the dietary effect on glycaemic and insulinemic responses at different time points post-prandial.

The glycaemic (GI) and insulinemic (II) index were calculated according to the following equations:

$$ GI = \frac{Area\, under \,570\,{\rm min}\; plasma \,glucose \,curve \,for \,meals (OB, BB, M)}{Area \,under \,570\,{\rm min}\; plasma \,glucose \,curve \,for \,H} \times 100 $$

$$ II = \frac{Area\, under \,570\,{\rm min}\;plasma \,insulin \,curve \,for \,meals (OB, BB, M)}{Area \,under \,570\,{\rm min}\;plasma \,insulin \,curve \,for \,H} \times 100 $$

### Statistical analyses

The effect of meals on post-prandial blood glucose and insulin concentrations were statistically evaluated as repeated measurements in a two-way ANOVA by use of the MIXED procedure in SAS. The calculated variables i.e. AUC_glucose/insulin_, GI, II, Peak_glucose/insulin_, Mean Time to Peak (MTP_glucose/insulin_) were the within-subject factors and the between-subjects’ factors were Meal and Time interval. Horse was included as random effect. The final model used was:

$$ {\text{Y}}_{\text{ij}} = \mu + \alpha_{\text{i}} + \beta_{\text{j}} + \varepsilon_{\text{ij}} $$

where Y_*ij*_ is the *ij*th predicted values, µ is the overall mean, α_*i*_ is the fixed effect of test meals (*i* = H, OB, BB, M), β_*j*_ is the fixed Time interval (*j* = 1, 2, 3, 4, 5) and ε_*ij*_ is the residual error. Time measurements were aggregated into 5 intervals (1 = 0–60 min, 2 = 60–120 min, 3 = 120–180 min, 4 = 180–330 min and 5 = 330–570 min) to measure the meal effect on calculated variables within each interval using the same model.

Pearson correlation coefficients were calculated for selected traits by use of the CORR procedure in SAS. Results were considered significantly different when *P* < 0.05 and a tendency when *P* < 0.10. The SAS version 9.2 [8] was used for all analyses.

## Results

### General health

All horses remained healthy throughout the experiment and showed no signs of discomfort due to repeated blood sampling. During adaptation periods, the horses were let out daily for about 3 h on a dirt paddock in groups of four, in order to satisfy their need for social interaction.

### Dietary composition and nutrient intake

Composition of meals and intake of carbohydrates are seen in Table 1. None of the meals exceeded the intended upper maximum limit of 540 g sugar and starch in total.

The effect of meals on plasma glucose (Table 2) and insulin (Table 3) concentration was measured and variables calculated. No significant difference was measured between these factors. Time to peak_glucose_ occurred 2–3 h after feeding and was numerically faster for the OB, BB and M meals as compared to the H meal.

**Table 2:** LSMeans of area under the plasma glucose curve (AUC), LSMean of peak plasma glucose (mmol/L), LSMeans of time to peak plasma glucose (min) and LSMeans of glycaemic index (%) for the different test meals

Test meals	AUC	Peak glucose	Time to glucose peak	GI
H	3101	5.9	206	100
OB	3152	6.3	131	102
BB	3140	6.7	135	102
M	3275	6.4	169	106
SE	166	0.4	40	5.2
*P* value	>0.10	>0.10	>0.10	>0.10

*LSMeans* least squares means, *SE* standard error, *H* Hay, *OB* Oats and Betfor^®^, *BB* Barley and Betfor^®^, *M* Equigard^®^

**Table 3:** LSMeans of area under the plasma insulin curve (AUC), LSMeans of peak plasma insulin (µU/ml), LSMeans of time to peak plasma insulin (min) and LSMeans of insulinemic index (%) for the different test meals

Test meals	AUC	Peak insulin	Time to insulin peak	II
H	6670	15.5	350^a^	100
OB	9296	27.2	105^b^	140
BB	8055	22.9	98^b^	121
M	8334	19.5	188^b^	125
SE	1186	2.8	35	18
*P* value	>0.10	0.07	<0.01	>0.10

*LSMeans* least squares means, *SE* standard error, *H* Hay, *OB* Oats and Betfor^®^, *BB* Barley and Betfor^®^, *M* Equigard^®^. Within a column, means without a common superscript ^a–b^ differ (*P* value < 0.05)

As expected the plasma insulin concentration followed the increase in plasma glucose concentration, however, no significant differences between meals were measured. Peak_insulin_ tended to differ significantly between meals, where the OB and BB meals resulted in the highest insulin concentration followed by the M meal. Peak_insulin_ occurred between 1.5–3 h after feeding of the OB, BB and M meals, which was significantly (*P* < 0.01) faster as compared to the H meal.

A significant effect of meal on plasma glucose and insulin concentration was measured when grouping time into intervals (Table 4). Meals significantly affected plasma insulin concentration 60–120 min post prandial, where the OB meal had the highest concentration followed by the BB, M and H meals. During the same time interval, the glucose concentration remained unaffected by meals. During the 120–180 min interval, insulin concentration was significantly (*P* < 0.01) higher on the M and OB meals as compared to the BB and H meals. The insulin concentration had decreased in the 180–330 min interval; however, it was still significantly (*P* = 0.01) higher than the H meal.

**Table 4:** LSMeans of plasma glucose (GLU, mmol/L) and insulin (INS, µU/ml) concentrations at five different time intervals (min) post prandially for the different test meals

Test meals	Time intervals									
	0–60		60–120		120–180		180–330		330–570	
	GLU	INS	GLU	INS	GLU	INS	GLU	INS	GLU	INS
H	5.3	11.5	5.7	15.1^c^	5.2^b^	10.9^b^	5.3	14.6^b^	5.4	11.3
OB	5.6	15.9	6.0	26.4^a^	5.1^b^	20.6^a^	5.7	17.4^a^	5.2	12.1
BB	5.3	14.6	6.0	20.9^b^	4.7^b^	14.1^b^	5.4	15.8^ab^	5.2	9.9
M	5.8	15.1	6.3	16.4^c^	5.9^a^	19.3^a^	5.9	17.4^a^	5.6	12.2
SE	0.3	2.2	0.3	3.1	0.4	2.1	0.2	2.2	0.2	2.0
*P* value	>0.1	>0.1	>0.1	<0.01	0.05	<0.01	>0.1	0.01	>0.1	>0.1

*LSMeans* least squares means, *SE* standard error, *H* Hay, *OB* Oats and Betfor^®^, *BB* Barley and Betfor^®^, *M* Equigard^®^. Within a column, means without a common superscript ^a–c^ differ (*P* value < 0.05)

No significant correlation was found between different carbohydrate fractions and glycaemic and insulinemic responses and calculated response variables in the horses.

## Discussion

The focus of this study was to measure the postprandial effect of various dietary carbohydrate compositions on blood plasma glucose and insulin concentration in horses. The meal effect on glycaemic and insulinemic response followed the expected pattern, where plasma concentrations increased after feeding and declined after peak concentration in agreement with the 5–6 h [9]. This fluctuation demonstrates an effective down regulation of plasma glucose by insulin [10], which indicates normal regulating pathways in healthy horses [11].

Insulin secretion is a biphasic response. The first phase secretion is rapid and lasts for 3–4 min followed by a second phase, which develops slowly and lasts longer [10]. The first phase secretion most likely resembles the peak_insulin_ response measured after 98–105 min (OB and BB meals) in the present experiment.

Several factors may affect and confound glycaemic and insulinemic responses in horses including rate of intake, meal size and feedstuff combination, absorption and perhaps gastro- intestinal passage rate [9]. No difference between test meals was measured on glycaemic and insulinemic responses in the present experiment. The sugar associated with the OB, BB and M meals was more easily accessible due to spray-on molasses, whereas sugar in hay is imbedded in the cell content [1] and therefore assumed less available for small intestine absorption. Additionally, the OB and BB meals contained pre-caecal digestible starch [2], which together with sugar is absorbed in the small intestine.

The M meal was associated with sugar intake but plasma glucose did not reach the highest level until the 120–180 min interval. This indicates a delayed glucose and subsequently insulin response perhaps due to slower gastric emptying leading to delayed glucose absorption. In other species diets high in soluble fibres have been shown to increase luminal viscosity [12] and thereby influencing the rate by which glucose is absorbed as well as the apparent insulin production. The viscosity of a product increases with increased molecular weight [13] and the luminal viscosity increases with increased intake of soluble NSP [14]. Caecal pH starts to decrease 3 h post prandially [2] due to starch reaching the hindgut when feeding the same meals as in the present study. This indicates that small intestine starch digestibility and glucose absorption takes place before 180 min post prandially in agreement with the present results. The horses in the present experiment were fed an additional hay meal 2½ h after the morning test meals, which presumably did not have had any negative effect on the post prandial metabolic responses as the small intestine absorption had already occurred. The blood plasma glucose response to all test meals was surprisingly low and resembled roughage only fed horses [15]. The M meal contained higher starch and sugar than the H meal, but less than BB or OB.

The GI and II predict the ranking of the glycaemic and insulinemic potential of different meals in horses. In the present study, the intention was to measure how various carbohydrates including fibre fractions affected the glycaemic and insulinemic responses. It therefore made sense to choose hay as reference when calculating the glycaemic and insulinemic indexes as hay or grass is the natural feed ingredient for horses [1] and then estimate the meal effect on GI and II in horses relative to hay. The method used in horses is not standardised and is therefore associated with errors [9] e.g. various length of the blood sampling interval, which consequently affects the calculation of the area under the glucose and insulin plasma curve. The GI and II are biased by long sampling intervals. It was not possible to determine, whether the effect on plasma glucose concentration was associated with absorption from the small intestine.

## Conclusions

Meals with different fibre composition did not affect the glycaemic- and insulinemic index in horses when fed at the levels used in the present experiment.
